# How different types of financial service providers support small- and medium- enterprises under the impact of COVID-19 pandemic: from the perspective of expectancy theory

**DOI:** 10.1186/s11782-020-00095-1

**Published:** 2020-12-22

**Authors:** Hua Song, Yudong Yang, Zheng Tao

**Affiliations:** Business School, Renmin University of China, Beijing, 100872 China

**Keywords:** 2019 novel coronavirus disease (COVID-19) pandemic, Financial service providers (FSPs), Expectancy theory, Financing to small- and medium-sized enterprises (SMEs)

## Abstract

The 2019 novel coronavirus disease (COVID-19) pandemic has significantly impacted several aspects of the society and the economy. A problem that needs prompt attention in this situation is the increasing difficulties faced by small- and medium-sized enterprises (SMEs) in raising capital, which has aroused great concern from multiple stakeholders such as public administrations and regulators. As the major supply of capital, financial service providers (FSPs) play a critical role in financing SMEs. However, how FSPs deal with SME financing during shocks has not yet been fully researched. Accordingly, in this study, a theoretical framework based on expectancy theory is proposed to explore the expected strategic adjustments of FSPs in financing SMEs. Specifically, this study investigates 272 FSPs in China on their expectancy and attitude on financing to SMEs during the COVID-19 pandemic. Furthermore, this study has divided FSPs into three categories: commercial banks, non-bank financial institutions, and credit-enhanced FSPs. Differences among these categories are compared and analyzed.

## Introduction

The 2019 novel coronavirus disease (COVID-19) pandemic has caused businesses to stagnate and disrupted supply chains, forcing numerous enterprises, especially small and medium-sized enterprises (SMEs), and individuals facing great pressure in terms of capital shortage (Guo et al. 2020). Multiple stakeholders, such as public administrations and regulators, have taken different measures to support SMEs financially. Public administrations and regulators, for example, have issued a series of supporting policies, including encouraging financing institutions to increase their overall capital supply with lower interest rates. Existing efforts, however, still seem to take little effect. Some studies have shown that the cash flow pressure of SMEs has not been significantly relieved even after the implementations of self-help measures and external supports (Bartik et al. 2020). Zhu et al. (2020) have found that over 70% of surveyed SMEs’ cash flow pressure has not been relieved significantly. Thus, it is necessary to turn the institutional and research focus to another vital stakeholder—financial service providers (FSPs).

Often, FSPs refer to organizations which provide financial services, especially those relevant to financing (Martin and Hofmann 2017; Silvestro and Lustrato 2014), including institutions that directly provide capital for companies—typically commercial banks, and providers who undertake information gathering and risk mitigation to facilitate financing activities like online industrial platforms and logistics service provider (Gelsomino et al. 2016; Hofmann 2009). As a significant external financing supplier to companies, FSPs play an essential role in dealing with capital shortages in SMEs (Song et al. 2018). Therefore, we explore how FSPs have considered supporting SMEs and making corresponding adjustments in financing SMEs under the impact of the pandemic.

However, these issues have not been explored yet. The existing literature on financing support for SMEs from FSPs primarily focus on the innovations in the financing modes (Abbasi et al. 2018; Lekkakos and Serrano 2016) and emerging technologies (Du et al. 2020; Hung et al. 2020), assuming that the external environment is relatively stable, while FSPs mostly consider their economic return. Even though it is foreseeable that the COVID-19 pandemic will significantly change the practices of FSPs (Goodell 2020), as the business environment deteriorates, FSPs tend to perform more social responsibilities to support SMEs (Talbot and Ordonez-Ponce 2020). Research has also explored FSPs’ strategic responses to external shocks, such as natural disasters and financial crises (Cortés and Strahan 2017; Dia 2013). However, few researchers focus on how FSPs respond in terms of SME financing. Moreover, in the wake of the COVID-19 pandemic, research has focused on the SMEs’ challenges regarding cash flow and their appeals, while ignoring the necessity of specific investigations concerning the financial providers (Bartik et al. 2020; Zhu et al. 2020). Nonetheless, a few works have preliminarily analyzed the potential impact of the pandemic on FSPs (Nicola et al. 2020; Yang et al. 2020) or qualitatively discussed how FSPs should support SMEs (Zhu et al. 2020), while leaving the real attitude and willingness of FSPs toward SME financing unexplored (Goodell 2020).

We propose a theoretical framework based on expectancy theory to explore how FSPs consider supporting SMEs, to address this gap. We conduct a targeted survey of 272 FSPs in China to provide relevant evidence. Furthermore, diverse types of FSPs with respective advantages behave differently and provide differentiated financing services. Thus, considering different kinds of FSPs is reasonable in this research (Martin and Hofmann 2017; Song et al. 2018) we further explore the differences in the anticipation among the different types of FSPs when financing SMEs under the impact of the pandemic, to elaborate the findings more accurately. FSPs are classified into three types: commercial banks, non-bank financial institutions, and credit-enhanced FSPs in this research.

This study contributes to existing research in the following ways. First, the theoretical framework is based on expectancy theory. We conduct surveys to explore the antecedents of real anticipation of FSPs in financing SMEs under the impact of the COVID-19 pandemic. This provides a more comprehensive insight into how FSPs support SMEs in such situations. Based on the literature review, this study is also the first to explore how to deal with the financing problems of SMEs in the context of the COVID-19 pandemic from the perspective of FSPs. Second, this study further identifies the differences in anticipation among different types of FSPs in the financing of SMEs, which provides more evidence to the research toward the differentiated role and orientation of distinctive FSPs. Third, applying expectancy theory to analyze the potential behavioral decisions of FSPs in financing SMEs widens the application range of this classical theory.

The remainder of the paper is organized as follows. Section 2 presents a literature review and proposes the research framework. Section 3 details the survey design and describes the data processing and research methods. Section 4 explores the influencing factors explicitly toward the anticipation of FSPs in financing SMEs under the impact of the economic shock of the pandemic. Further, we compare and analyze the differences among distinctive FSPs. Section 5 presents the conclusion and provides some suggestions for how FSPs support SMEs in financing.

## Literature review and theoretical framework

### FSPs’ financing to SMEs

The high-cost and slow financing of SMEs is a great challenge for economic development, which has aroused the concerns of many scholars (Song et al. 2018). With high operational uncertainty, SMEs lack sufficient collateral and guarantees. Their financial statements are weak and unreliable, making it difficult and costly for FSPs to evaluate their credit status and the corresponding default risk. As borrowers, SMEs usually have better information than FSPs of their transaction motive and loan repayment ability, which causes severe ex-ante information asymmetry between SMEs and FSPs (Berger and Udell 2006; Roberts 2015). Moreover, SMEs lack well-established corporate governance, their informatization capacity is weak, and their supervision after signing a financing contract is not effective, resulting in an ex-post information asymmetry (Berger and Udell 2006; Song et al. 2020). FSPs are reluctant to provide financing for SMEs due to the high information asymmetry. Even if FSPs are willing to provide funds, they often demand high-interest rates to compensate for possible default risk.

With the growing financing demands of SMEs, FSPs gradually focus on mitigating the information asymmetry to extend the lending business toward SMEs. The extant research has explored how FSPs support SMEs in addressing their financing problems through innovative financing modes and emerging technology (Abbasi et al. 2018; Du et al. 2020; Lekkakos and Serrano 2016). Specifically, FSPs innovate the financing modes by collaborating with other stakeholders, such as supply chain participants, to collect information to replace traditional lending. For example, supply chain finance is widely considered a new financing approach for SMEs by FSPs (Abbasi et al. 2018; Hofmann 2009). Recently, another financial innovation instrument, bank-tax interaction, has been adopted by some FSPs to provide financing for SMEs based on historical information of their tax payments, supplied by public administrations (Luo et al. 2020).

Moreover, emerging information technologies such as the internet of things (Abbasi et al. 2018), blockchain (Chod et al. 2020; Du et al. 2020), and big data (Hung et al. 2020), are applied to mitigate information asymmetry by improving the range, richness, and quality of information about SMEs. However, most studies aim to offer innovative financing approaches with an underlying assumption that the economic and business environment is relatively stable (FSPs are willing to support SMEs under such conditions). Only a few studies focus on the potential changes when encountering external shocks such as natural disasters, pandemics, among others (Cortés and Strahan 2017; Dia 2013). Nevertheless, the research findings regarding the response of FSPs to the external shocks are inconsistent. Some scholars have found that FSPs may reduce the provision of financing and investments in response to adverse shocks, considering the potential economic loss caused by the increased default risk (Dia 2013; Gong et al. 2020). However, it has also been established that FSPs could actively support clients as their social responsibility (Cortés and Strahan 2017; Talbot and Ordonez-Ponce 2020). Besides the inconsistent findings, most relevant studies ignore the support provided by FSPs to specific clients, especially SMEs facing severe pressure from the shortage of capital.

Research gaps also exist in the context of the COVID-19 pandemic. SMEs lacking the ability and resources to cope with uncertainties are more vulnerable to risks like the COVID-19 pandemic, causing severe capital shortage (Zhu et al. 2020). Consequently, FSPs may significantly change their attitude toward financing SMEs and make corresponding strategic adjustments in response to the pandemic (Goodell 2020). However, to this date, existing research has only qualitatively discussed how other stakeholders enhance FSPs’ willingness to provide financial support to SMEs (Bartik et al. 2020), leaving the real attitude and expectancy of FSPs toward financing SMEs unexplored, especially the trade-off made by the FSPs between economic performance and social responsibility in such a difficult period (Gong et al. 2020).

### Expectancy theory and theoretical framework

Expectancy theory is one of the motivation theories, predicting that actions are driven by two factors: expectation (expectancy) which is the probability that effort will contribute to achieving expected goals, and the perceived value (valence) of the outcome arising from the actions. This is also known as the “expectancy-valence” framework (Snead and Harrell 1994; Vroom 1964). Initially, expectancy theory mainly explains an individual’s behavioral intentions in various fields, such as employee motivation and organizational behavior (Chen and Fang 2008; Fudge and Schlacter 1999). With increasing development and application, this theory provides insights into the decision-making process needed to achieve goals in an organization, including exporting strategy (Wood et al. 2015), supplier development (Chen et al. 2016), among others.

The review of expectancy theory suggests that it can be applied to effectively explore the decisions on adjustments made by FSPs to finance SMEs in the face of the pandemic and evaluate the potential outcome arising from the change. Unlike most research, which apply the “expectancy-value” framework to explore the relationships between the determinants, effects, and purposes, this study aims to predict or portray FSPs’ potential behaviors in financing SMEs according to the two types of determinants, and to discuss the corresponding outcome. Specifically, we suggest that financing expectancy and financing valence of FSPs affect their financing of SMEs. Financing expectancy refers to FSPs’ perceived probability that the effort can provide financing to SMEs and yield the desired outcome. Financing valence is defined as the FSPs’ perceived value of the desired outcome, including economic performance and social responsibility in this research.

The existing research has identified several levels of factors that could impact the expectancy and valence, including individual-, activity-, firm-, interfirm- and environment-level factors (Chen et al. 2016). Thus, following the framework proposed by Chen et al. (2016), we explore the FSPs’ expectancy and valence toward financing SMEs by investigating such levels of determinants. According to the review of expectancy theory and the literature on FSPs’ financing SMEs, this study considers the effects of firm-level factors such as the relevant resources and capabilities, interfirm-level factors such as collaboration with peer FSPs’ focal firm, and institution, or environment-level factors such as collaboration with public administrations and regulators on the “financing expectancy,” and the activity-level factors which reflect FSPs’ preferences for the outcome obtained by providing financing to SMEs on the “financing valence.”

#### Financing expectancy

First, at the firm-level, the size of the FSPs’ financial resource is positively associated with the ability to withstand external risks (Beck 2013). Extant research demonstrates that the innovative financing modes and emerging information technologies could help mitigate information asymmetry, which is much dependent on the relevant informatization capability and human resource of professionals (Abbasi et al. 2018; Gomm 2010; Sheng 2020), thus, making it difficult for FSPs that lack corresponding resources and capabilities to support SMEs. Second, at the interfirm-level, focal firms, as the business organizer and coordinator of the whole supply chain, play an essential role in transmitting information of SMEs to FSPs (Lekkakos and Serrano 2016; Wu et al. 2019). Therefore, lack of collaborations with focal firms may increase difficulties in FSPs’ financing to SMEs. Moreover, existing studies have highlighted the advantages of collaboration between different types of FSPs (Ntwiga 2020; Zhu et al. 2015). For instance, collaboration among FSPs can aggregate abundant financial resources, increasing the financing supply, and decreasing the financing cost due to the “scale effect” (Clark et al. 2018). FSPs could also enhance their risk appetite and reduce overall financing risk through interaction with other FSPs like insurance companies (Mäenpää and Voutilainen 2011). Thus, it is more feasible for FSPs to finance SMEs when supported by other partners. Third, at the institution- or environment-level, policies and regulations tend to be issued frequently to cope with external shocks like the pandemic, which often greatly influences FSPs to support SMEs (Zhang et al. 2020; Zhu et al. 2020). Some studies have shown that public administrations and regulators may help FSPs increase liquidity provision, risk tolerance, deal with bad debts, and many more (Zhu et al. 2020). The institution-level factors like support from public administrations and regulators are positively associated with FSPs’ expectancy in financing SMEs.

#### Financing valence

Considering that FSPs’ perceived value of performance arising from the financing action is closely related to characteristics of the act itself, we mainly discuss the activity-level factors in this study. First, financing volume, financing rate, and default rate are essential elements of financing actions that determine the financial performance of both the borrower and lender (Gomm 2010; Ongore and Kusa 2013). As the financing volume increases, so does the business scale of FSPs, and SMEs can receive more financing to cope with the crisis caused by the pandemic. However, the effects of the financing rate on FSPs and SMEs are contradictory. A higher financing rate means that FSPs could realize more profit as risk compensation, while the cost of financing for SMEs will increase correspondingly. Besides, a high default rate may increase the bankruptcy risk of FSPs (Ongore and Kusa 2013). Second, recent research has shown that the COVID-19 pandemic has had a varied impact on industries (Bartik et al. 2020; Ding et al. 2020). Thus, financing demands and default risks also vary by sector. FSPs financing firms under distress also face losses. At the same time, FSPs offer hope by supporting SMEs in such a turbulent time and act with the motive of social responsibility (Bartik et al. 2020; Talbot and Ordonez-Ponce 2020). Another factor is the type of financing products provided by FSPs. Traditional products, including fixed asset mortgage and third-party guarantee, are relatively secure for most FSPs in controlling the financing risk. However, it is difficult for SMEs that lack mortgage collateral or guarantors to meet the corresponding requirements (Beck 2013). The innovative financing modes like reverse factoring help SMEs get access to financing. However, their implementation demands FSPs to invest more resources in informatization construction and collaboration with multi-stakeholders (Lekkakos and Serrano 2016).

**Fig. 1:**
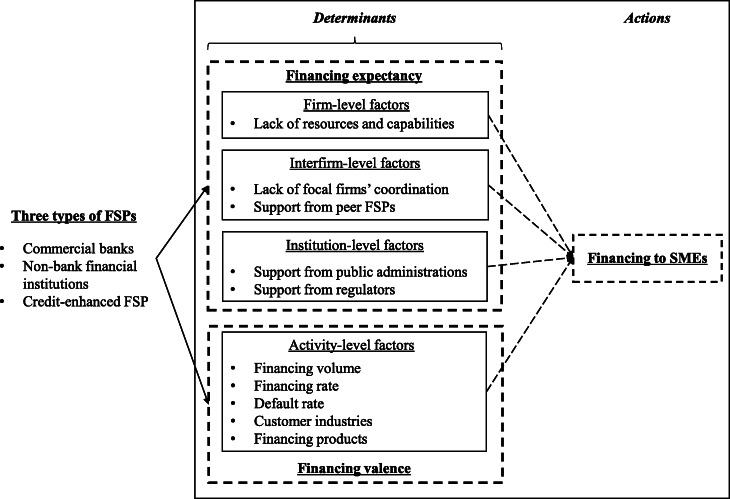
Theoretical framework *Notes.* The dotted line indicates that the relationship has not been empirically tested in this research

Based on the review and analysis, we present a theoretical framework in the “*Determinants*” and “*Actions*” parts of Fig. 1. The framework explores how FSPs consider conducting adjustments in supporting the financing of SMEs (action), and the corresponding performance would be achievable. It also needs to be emphasized that this study does not directly measure financing expectancy, financing valency, and financing to SMEs, but analyzes and forecasts them through the important antecedents mentioned.

### Different types of FSPs

FSPs traditionally refer to banks and non-bank financial institutions, such as guarantee companies and factoring companies (Silvestro and Lustrato 2014). As information is increasingly important to financial services, a growing number of service providers, such as fintech companies, service providers of supply chain management, and industrial platform companies, provide financial services and help financial institutions gather information and control risk (hereafter “credit-enhanced FSPs”) (Leyer et al. 2016; Martin and Hofmann 2017; Sheng 2020). Recently, some scholars have expanded the definition of FSPs to include all critical participants in providing financial services (Ma et al. 2020).

Some scholars have established that different FSPs with respective advantages play various roles in financing SMEs (Martin and Hofmann 2017; Song et al. 2018). For instance, compared with traditional FSPs—financial institutions such as commercial banks—logistics service providers promptly gather more valid information about logistics elements such as inventory, which could reduce transaction costs and default risks (Hofmann 2009). Supply chain service providers can effectively reduce information asymmetry between supply chain participants and financial institutions through the integrated management of supply chain flows (Martin and Hofmann 2017). Fintech service providers are capable of operating financial businesses that are underserved by traditional banks (Ntwiga 2020). Furthermore, among financial institutions, commercial banks possess abundant financial resources and serve more enterprises, the key financing source of SMEs (Song et al. 2018). Thus, it is necessary to distinguish commercial banks from the non-bank financial institutions such as small-loan companies, factoring companies, and guarantee companies.

Given the differentiated roles and purposes, this study classifies FSPs into three types: commercial banks, non-bank financial institutions, and credit-enhanced FSPs. It is expected that they will behave differently under the impact of the pandemic. However, how they each respond and the specific differences between them have not been fully explored yet. Moreover, the increased uncertainty during this period has caused difficulty in predicting the attitude and behaviors of FSPs. For instance, commercial banks possess more resources than credit-enhanced FSPs. They have an advantage in information gathering and processing over others (Martin and Hofmann 2017; Song et al. 2018). Consequently, will the financing expectancy of commercial banks be influenced more by the lack of informatization capability under the impact of the pandemic? Will the expectancy of credit-enhanced FSPs be affected significantly by the lack of other resources like financial resources?

In conclusion, to better understand the financing attitude and willingness of different FSPs, this study explores the possible differences in the determinants and actions among three types of FSPs, enriching the theoretical framework, shown in Fig. 1.

## Research design, methodology, and sample description

### Survey design and data collection

In this study, an online survey is conducted to obtain research data. Based on the theoretical literature, to formulate the initial scale, we invite academic experts and professional executives to develop the questionnaire to ensure its validity and effectiveness. Specifically, some indicators are measured directly based on existing literature and authoritative standards. For example, the items of *total assets in the last fiscal year* are designed according to *Standard Provisions on the Classification of Financial Enterprises* from the People’s Bank of China. The items of *changes in financing volume* are developed by referring to the measurement of financial performance in Gomm (2010) and Lu et al. (2020). Meanwhile, some items of the indicators are proposed directly through development and scrutiny by experts. Regarding *the number of SMEs served in the last fiscal year*, the specific items like “A. < 100” and “B. 100–1000” are developed according to experts’ experience. Elements of the other indicators are developed by combining the literature with industrial experience. *Lack of internal resources and capabilities*, for instance, has been discussed in many studies (e.g., Abbasi et al. 2018; Beck 2013), while the items to measure for each indicator have not been specified yet. Thus, combined with industrial experience, experts have independently listed the relevant examples. We select the most representative statements to develop the specific item of each indicator. An item is adopted if and only if all the experts accept it. Before the formal release of the questionnaire, we conduct a pilot survey and revise it to ensure the validity of items.

This questionnaire adopts various measurement methods for different research purposes, including a single choice or 5-point Likert Scale, multiple-choice, and ranking method. The entire survey consists of three parts: sample characteristics, financing expectancy, and financing valence. In the sample characteristic part, we require respondents to choose an option in each indicator to identify specific characteristics of FSPs. In the financing expectancy part, there are several items in each indicator. We mainly focus on whether a particular item impacts FSPs; thus, a multiple-choice method is designed to measure the indicators, effectively reducing response time. In the financing valence part, we use 5-point Likert Scale to measure the expected changes in financing volume, financing interest rate, and default rate under the impact of the pandemic. Further, considering that FSPs serve several client industries and provide more than one financing products, we adopt the ranking method to explore the differences in FSPs’ preference toward diverse client industries and financing products.

More details about the survey development process and the results of the questionnaire are provided in the Appendix.

The questionnaire is formally distributed and collected between February 17 and March 3, 2020. During this period, there was widespread stagnation of businesses and disruption of the supply chain, reflecting the peak of the epidemic’s impact in China. Parallelly, it was also a critical period for multi-stakeholders to participate in the prevention and control of the pandemic and the resumption of enterprise operations. Thus, this period was appropriate for exploring the FSPs’ response.

We use a snowball sampling approach to obtain data through personal relations from industry associations, such as the finance committee of the China Federation of Logistics & Purchasing, and randomly sent questionnaires. All the respondents are medium- or top-level executives of the institution, including bank presidents, CEOs, and financial managers. To collect information from FSPs, we contact each respondent, explain the purpose of the survey, and confirm their responses. In total, 312 questionnaires are distributed, of which 272 samples are valid and retained; the rate of valid samples is 87.18%. 

### Research methods

The analysis is divided into two parts. First, we adopt a descriptive statistics analysis to demonstrate the results of FSPs’ financing expectancy and valence. Second, to further explore the differences in FSPs, the non-parametric hypothesis analysis method is adopted. Furthermore, as the distribution of samples and the assumption of the homogeneity of variances are unknown in this study, the method is more appropriate because it does not require assumptions to be made about data parameters and is widely used in business research (Simar and Wilson 2002). Specifically, we use the Wilcoxon rank-sum test (also called the Mann-Whitney U test) to compare the differences between two independent samples from the same population. Further, we use the Kruskal-Wallis rank-sum test to compare the differences between multiple (more than two) independent samples from the same population. Finally, we use the Wilcoxon matched-pairs signed-ranks test to compare the differences between the two dimensions of the same sample. The non-parametric hypothesis analysis is conducted using the SPSS 23.0 software.

### Sample characteristics

#### Total assets in the last fiscal year

In terms of the assets of the surveyed FSPs, 14.0% have more than RMB100 billion, most of these are commercial banks, 14.7% between RMB5 billion and RMB100 billion, 19.1% between RMB1 billion and RMB5 billion, and 52.2% less than RMB1 billion.

#### Financing volume to SMEs in the last fiscal year

Financing volume here refers to not only the scale of capital directly provided by financial institutions, but also the scale of funds indirectly obtained by credit-enhanced FSPs for SMEs in the last fiscal year (2019).

Among the FSPs, 6.3% provided more than RMB100 billion, and mostly are commercial banks and fintech service providers. The remaining included 13.2% which provided more than RMB10 billion and less than RMB100 billion, 25.0% which provided more than RMB1 billion and less than RMB10 billion, 26.5% which provided more than RMB100 million and less than RMB1 billion, and 29% which provided less than RMB100 million.

#### Business types

In terms of business types, we first classify all samples into two categories of FSPs (financial institutions and credit-enhanced FSPs), based on whether the sample institution is licensed to engage in direct lending, and explore the differences between them. Furthermore, we also differentiate commercial banks from non-bank financial institutions. According to the *Notice on Regulating Inter-Bank Business of Financial Institutions (2014)*, jointly issued by the People’s Bank of China and the China Banking Regulatory Commission, non-bank financial institutions consist of companies which are trusts, small-loan lender, funds, guarantors, financial holdings, among others.

**Fig. 2:**
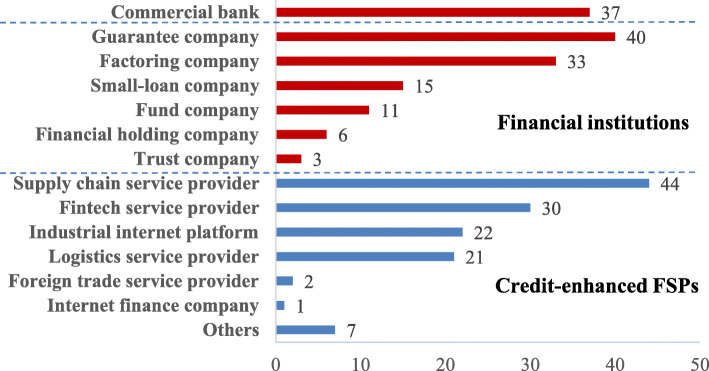
Distribution of business types in the sample

Among the 272 FSPs that respond to the survey, 53.3% are financial institutions that provide funds directly, of which 13.6% are commercial banks, 14.7% are guarantee companies, and 12.1% are factoring companies; 46.7% are credit-enhanced FSPs that assist financial institutions to provide financial services for SMEs, among which 16.2% are supply chain service companies, 11.0% are fintech service providers, and 8.1% are industrial internet platforms. The specific data regarding each type of FSP are shown in Fig. 2.

#### Differences in sample characteristics between diverse types of FSPs

As shown in Table 1, there are significant differences between the three types of total assets and financing volume (*p* < 0.001). Specifically, the average score of the assets of commercial banks is significantly higher than that of the non-bank financial institutions (7.46 > 3.00, *p* < 0.001) and credit-enhanced FSPs (7.46 > 2.76, *p* < 0.001). Although the average score of the assets of the non-bank financial institutions is marginally higher than that of credit-enhanced FSPs, the difference between them is not statistically significant (3.00 > 2.76, *p* = 0.289). Similarly, the average score for the financing volume of commercial banks is significantly higher than that of the non-bank financial institutions (5.28 > 2.61, *p* < 0.001) and credit-enhanced FSPs (5.28 > 2.48, *p* < 0.001), and there is no significant difference between the non-bank financial institutions and credit-enhanced FSPs (2.61 > 2.48, *p* = 0.169).

**Table 1:** Total assets and financing volume of different business types

	Financial institutions		Credit-enhanced FSPs (C)	Pairwise comparison
	Commercial banks (A)	Non-bank financial institutions (B)		
Total assets (*p* < 0.001)	7.46	3.00	2.76	A:B^***^
				A:C^***^
				B:C
Financing volume (*p* < 0.001)	5.28	2.61	2.48	A:B^***^
				A:C^***^
				B:C
Number	37	108	127	

*Notes.* The *p* values in the first column refer to the overall differences between the three types. The values in this table refer to the average score of the corresponding items in the Appendix of each type, reflecting the objective condition of surveyed FSPs. The higher the value, the higher is the total assets or financing volume in the last fiscal year

*** *p* < 0.001, ** *p* < 0.01, * *p* < 0.1

The results show that in comparison with other FSPs, commercial banks hold several financial resources and are the main forces that provide financing to SMEs.

## Data analysis and findings

### Financing expectancy

**Fig. 3:**
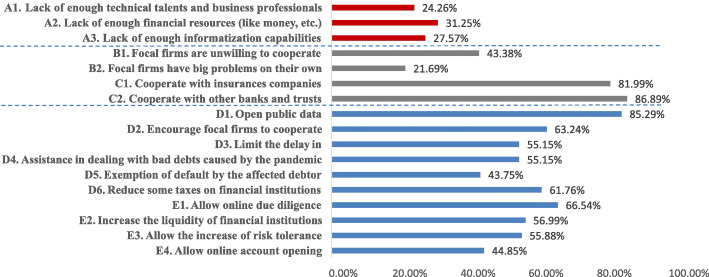
Distribution of factors in financing expectancy

We develop several items to evaluate FSPs’ financing expectancy to finance SMEs from three perspectives or levels: firm-, interfirm- and institution-level. We then ask the respondents which items are in line with their company’s situation when financing SMEs in the face of the pandemic. The respondents could make multiple choices. The specific results are shown in Fig. 3. Further, we explore the differences in these three levels among the diverse types. The results are listed in Tables 2, 3, and 4.

**Table 2:** Differences in firm-level factors among diverse types

	Financial institutions		Credit-enhanced FSPs (C)	Pairwise comparison
	Commercial banks (A)	Non-bank financial institutions (B)		
A1 (*p* = 0.460)	0.32	0.23	0.23	
A2 (*p* < 0.001)	0.08	0.26	0.43	A:B
				A:C^***^
				B:C^*^
A3 (*p* = 0.006)	0.49	0.27	0.22	A:B^*^
				A:C^***^
				B:C
Number	37	108	127	

*Notes.* The *p* values in the first column refer to the overall differences between the three types. The values in the table are the ratio of the samples which select the item

*** *p* < 0.001, ** *p* < 0.01, * *p* < 0.1

**Table 3:** Differences in interfirm-level factors among diverse types

	Financial institutions		Credit-enhanced FSPs (C)	Pairwise comparison
	Commercial banks (A)	Non-bank financial institutions (B)		
B1 (*p* < 0.001)	0.70	0.45	0.34	A:B^*^
				A:C^***^
				B:C
B2 (*p* = 0.003)	0.43	0.19	0.17	A:B^**^
				A:C^**^
				B:C
C1 (*p* = 0.938)	0.83	0.82	0.82	
C2 (*p* = 0.022)	0.81	0.87	0.88	A:B^*^
				A:C^*^
				B:C
Number	37	108	127	

*Notes*. The *p* values in the first column refer to the overall differences between the three types. The values in the table are the ratio of the samples which select the item

*** *p* < 0.001, ** *p* < 0.01, * *p* < 0.1

**Table 4:** Differences in institution-level factors among diverse types

	Financial institutions		Credit-enhanced FSPs (C)	Pairwise comparison
	Commercial banks (A)	Non-bank financial institutions (B)		
D1 (*p* = 0.329)	0.89	0.88	0.82	
D2 (*p* = 0.082)	0.78	0.64	0.58	A:B
				A:C^*^
				B:C
D3 (*p* = 0.123)	0.65	0.59	0.49	
D4 (*p* < 0.001)	0.84	0.56	0.47	A:B^*^
				A:C^***^
				B:C
D5 (*p* = 0.001)	0.57	0.54	0.32	A:B
				A:C^*^
				B:C^**^
D6 (*p* = 0.017)	0.70	0.69	0.62	A:B
				A:C
				B:C^*^
E1 (*p* = 0.148)	0.78	0.61	0.68	
E2 (*p* = 0.054)	0.54	0.49	0.65	A:B
				A:C
				B:C^*^
E3 (*p* = 0.006)	0.68	0.64	0.48	A:B
				A:C^*^
				B:C^*^
E4 (*p* = 0.003)	0.65	0.34	0.48	A:B
				A:C^**^
				B:C^**^
Number	37	108	127	

*Notes*. The *p* values in the first column refer to the overall differences between the three types. The values in the table are the ratio of the samples which select the item

*** *p <* 0.001, ** *p <* 0.01, * *p <* 0.1

#### Firm-level factors

Among the three items of firm-level factors, lack of sufficient financial resources receives the most attention (31.25%), and the lack of informatization capabilities (27.57%) and talents and professionals (24.26%) are also important factors that prevent FSPs from providing financing. Overall, most FSPs’ financing expectancies are less affected by the constraints of internal resources and capabilities.

To ascertain the type of FSPs facing difficulty with these factors, we further explore the differences between the three types, and the corresponding results are listed in Table 2. The ratio at which credit-enhanced FSPs lack sufficient money is significantly higher than that of commercial banks (0.43 > 0.08, *p* < 0.001) and non-bank financial institutions (0.43 > 0.26, *p* = 0.019 < 0.1), while the difference between commercial banks and the non-bank financial institutions is not significant. Moreover, the ratio at which commercial banks lack informatization capabilities is significantly higher than that of non-bank financial institutions (0.49 > 0.27, *p* = 0.032 < 0.1) and credit-enhanced FSPs (0.49 > 0.22, *p* = 0.004 < 0.01), while the difference between non-bank financial institutions and credit-enhanced FSPs is not significant. There is no significant difference in the lack of professionals among the three types, though the ratio of commercial banks is relatively higher (0.32 > 0.23). According to the results, the strengths and weaknesses of different types of banks in financing SMEs differ.

#### Interfirm-level factors

In this part, we explore the impact of two critical stakeholders: focal firms and peer FSPs.

Firstly, from the results in Fig. 3, we find that nearly half of the surveyed FSPs are confronted with non-cooperative focal firms. However, focal firms also face challenges resulting from the pandemic; only 21.69% of FSPs accept this. Furthermore, based on the results shown in Table 3, the ratio of lack of collaboration between focal firms and commercial banks is as high as 70%, significantly higher than that of non-bank financial institutions (0.70 > 0.45, *p* = 0.0225 < 0.1) and credit-enhanced FSPs (0.70 > 0.34, *p* < 0.001). Most credit-enhanced FSPs, including industrial internet platforms and service providers of supply chain management, have close ties with focal firms and are familiar with critical information and data of supply chain operation. Focal firms, at critical positions of the supply chain, are linked to numerous SMEs in the upstream and downstream. Thus, they understand the real operational and financial status of SMEs. This helps FSPs make financing decisions and control risks (Caniato et al. 2016; Pfohl and Gomm 2009). However, focal firms have not always been reliable, and may also be the source of financing risks. Similarly, for the item on whether focal firms have suffered from great challenges under the impact of pandemic, the ratio of commercial banks is significantly higher than that of non-bank financial institutions (0.43 > 0.19, *p* = 0.007 < 0.01) and credit-enhanced FSPs (0.43 > 0.17, *p* = 0 .002 < 0.01). There is also no statistically significant difference between the other two types of FSPs.

Secondly, as shown in Fig. 3, the ratios of need for support from peer FSPs are markedly higher with values of both items above 80%, reflecting the strong demand for collaboration with peer FSPs in the face of the pandemic. Table 2b shows that all three types of FSPs claim a strong requirement for cooperation with insurance, and the percentages are both above 80%. Moreover, more than 80% of surveyed FSPs claim a need for cooperation with banks and trusts, while the ratio of commercial banks is slightly lower than that of non-bank financial institutions (0.81 < 0.87, *p* = 0.041 < 0.1) and credit-enhanced FSPs (0.81 < 0.88, *p* = 0.021 < 0.1). The results reflect that even commercial banks with abundant financial resources have great needs for collaboration with peer FSPs. Thus, most FSPs must cooperate with others to realize complementarities and promote common development.

#### Institution-level factors

At the institution level, we investigate the factors concerning support from public administrations and regulators. Figure 3 shows that most FSPs need support from public administrations in providing information, supporting FSPs’ crediting, and online due diligence. Similarly, we discuss the differences in these factors among diverse types of FSPs.

Regarding support from public administrations, commercial banks’ need is generally greater than that of the other two types; credit-enhanced FSPs seem to demand less. Specifically, commercial banks need focal firms to cooperate by confirming receivables and releasing useful information; thus, commercial banks’ requirement for public administration support is significantly greater than that of credit-enhanced FSPs (0.78 > 0.58, *p* = 0.078 < 0.1) as shown in Table 4. This is consistent with the conclusion discussed that commercial banks are confronted with difficulties concerning the lack of coordination of focal firms. There is no significant difference in the need to limit late payments between the three types of institutes, though the ratio of commercial banks is still the highest (0.65 > 0.59 > 0.49). Addressing the debts as a result of the pandemic, the needs of commercial banks are significantly greater than that of non-bank financial institutions (0.84 > 0.56, *p* = 0.009 < 0.01) and credit-enhanced FSPs (0.84 > 0.47, *p* < 0.001). This is consistent with the results regarding the expected changes in the default rate. Further, commercial banks have a greater need to exempt defaults by affected debtors than credit-enhanced FSPs (0.57 > 0.32, *p* = 0.020 < 0.1), the same applies to non-bank financial institutions (0.54 > 0.32, *p* = 0.002 < 0.01). Thus, we infer that commercial banks are confronted with high levels of risk of bad debts under the impact of the pandemic, and the non-bank financial institutions follow. The need to reduce taxes on FSPs is a widespread concern. This need is significantly less for credit-enhanced FSPs than that of non-bank financial institutions (0.62 < 0.69, *p* = 0.026 < 0.1), and relatively less than that of commercial banks (0.62 < 0.70) because of their indirect involvement in financing services.

The need for support from regulators is greater for commercial banks than non-bank financial institutions and credit-enhanced FSPs; non-bank financial institutions tend to demand less. More details regarding specific items are listed in Table 4. First, there is no significant difference in demand for online due diligence among the three types (*p* = 0.148), though the overall level is high. The isolation and travel restrictions resulting from the pandemic have made it difficult to conduct face-to-face operations, a significant challenge for many businesses. However, there are differences in other factors. The need for increased liquidity of FSPs, by reducing required reserve ratios and reverse repurchase is significantly greater for credit-enhanced FSPs than non-bank financial institutions (0.65 > 0.49, *p* = 0.051 < 0.1). However, the difference between them credit-enhanced FSPs and commercial banks is not statistically significant. The results are also consistent with the “firm-level factors” section that credit-enhanced FSPs face greater issues related to insufficient funds with providing financing. Concerning the need for increased risk tolerance, the responses of commercial banks and non-bank financial institutions are more significant than those of credit-enhanced FSPs (0.68 > 0.48, *p* = 0.055 < 0.1; 0.64 > 0.48, *p* = 0.015 < 0.1), reflecting the expected increase in default risk and insufficient risk control for financial institutions. Moreover, regarding online account opening, the requirement of commercial banks is greater than that of non-bank financial institutions (0.65 > 0.34, *p* = 0.004 < 0.01), and the need of credit-enhanced FSPs is significantly greater than that of non-bank financial institutions (0.48 > 0.34, *p* = 0.004 < 0.01). This may be because the non-bank financial institutions’ businesses, such as the factoring and bonding companies, are primarily based on existing customers and less dependent on obtaining new customers.

### Financing valence

#### Expected changes in financing volume, financing rate, and default rate

To explore the impact of the pandemic, we ask the respondents how the financing volume, financing rate, and default rate will change during or after the pandemic. The respondents are required to choose the item which fits most, including “A. comprehensively decrease, B. most decrease, some increase, C. remain unchanged, D. most increase, some decrease, and E. comprehensively increase.”

**Fig. 4:**
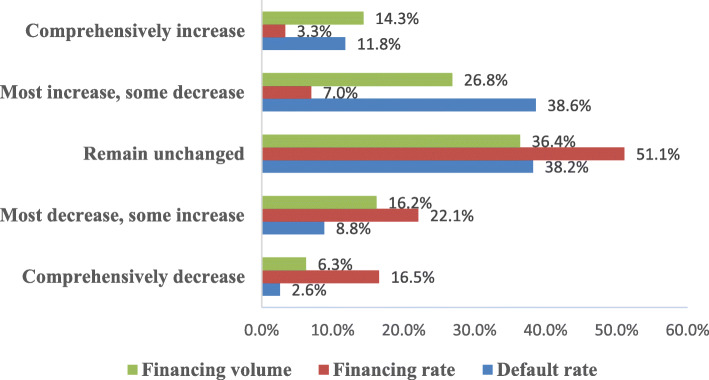
Expected changes in financing volume, financing rate, and default rate of all FSPs

As shown in Fig. 4, most FSPs increase their financing volume or remain unchanged, and most FSPs decrease their financing interest rate or remain unchanged. Besides, FSPs generally believe that the default rates of SMEs will rise under the impact of the pandemic. Based on the results, we conclude that despite increased risks, most FSPs are still willing to provide SMEs with more financing despite a lower economic income, reflecting that most FSPs undertake the social responsibility of supporting SMEs actively.

Furthermore, different FSPs also behave differently, and the results are shown in Table 5. Compared with the other FSPs, commercial banks provide more financing to SMEs and actively decrease the financing rate. As shown, there are significant differences in the changes of both financing volume and financing interest rate among the three types (*p* < 0.001), while there are no significant differences in the changes of default rate (*p* = 0.230 > 0.1). Specifically, the average score regarding expected changes of financing volume of commercial banks is significantly higher than that of the non-bank financial institutions (3.81 > 3.31, *p* = 0.023 < 0.1) and credit-enhanced FSPs (3.81 > 3.07, *p* < 0.001). However, the score of the non-bank financial institutions is not significantly different from that of credit-enhanced FSPs (3.31 > 3.07, *p* = 0.481 > 0.1). For expected changes of financing rate, the average score of commercial banks is significantly lower than that of the non-bank financial institutions (2.08 < 2.53, *p* = 0.017 < 0.1) and credit-enhanced FSPs (2.08 < 2.78, *p* < 0.001), and the score of the non-bank financial institutions is also significantly different from that of credit-enhanced FSPs (2.53 < 2.78, *p* = 0.032 < 0.1).

**Table 5:** Expected changes in financing volume, financing rate, and default rate for different business types

Expected changes in	Financial institutions		Credit-enhanced FSPs (C)	Pairwise comparison
	Commercial banks (A)	Non-bank financial institutions (B)		
Financing volume (*p* < 0.001)	3.81	3.31	3.07	A:B^*^
				A:C^***^
				B:C
Financing rate (*p* < 0.001)	2.08	2.53	2.78	A:B^*^
				A:C^***^
				B:C^*^
Default rate (*p* = 0.230)	3.59	3.55	3.39	
Number	37	108	127	

*Notes*. The *p* values in the first column refer to the overall differences between the three types. The values in this table refer to the average score of the corresponding items in the Appendix of each type, reflecting the willingness or attitude on the adjustment of financing strategies. The higher the value, the more the expected financing volume/financing rate/default rate will increase

*** *p <* 0.001, ** *p <* 0.01, * *p <* 0.1

This analysis shows that, when confronted with the systematic risks caused by the pandemic, different types of FSPs suffer from common pressures of rising default rates. However, commercial banks which possess abundant financial resources and customers will be more active in providing low-cost and accessible financing for SMEs.

#### Changes of served industries

We ask the respondents about the industry groups of the SMEs to which their company mainly provide financing services, before and after the pandemic, and ask them to use the ranking method to choose the item. Then, we compare the differences between served industries between the two phases through a non-parametric pairing test to explore the changes in FSPs’ propensity.

The corresponding results are shown in Tables 6 and 7, indicating that the importance of served industries has changed significantly due to the pandemic. Specifically, FSPs significantly increase their financing support to SMEs in the medical industry (the growth rate of the weighted score is 38.73%; *p* < 0.001), and also express interest in providing financing to SMEs in the public service, hospitality and tourism, education, business service, IT, and manufacturing industries. However, FSPs’ attitudes toward financing SMEs in the construction (*−*23.83%; *p* < 0.001), energy (*−*10.79%; *p* = 0.074 < 0.1) and trade industries (*−*6.37%; *p* = 0.065 < 0.1) is significantly negative, and FSPs also decrease their support to SMEs in the culture, agriculture, and transportation industries.

**Table 6:** Positive changes in financing attitude toward served industries before/after the pandemic

	IT	Business service	Education	Manufacturing	Medical	Hospitality and tourism	Public service
Before the pandemic	2.71	0.98	0.93	7.97	3.52	1.47	1.32
During and after the pandemic	2.87	1.04	1.05	8.04	4.89	1.66	1.62
Changes	5.83%	6.02%	12.60%	0.83%	38.73%	13.00%	22.56%
*p*-value	0.390	0.626	0.466	0.973	0.000^***^	0.224	0.105

*Notes*. *** *p <* 0.001, ** *p <* 0.01, * *p <* 0.1

**Table 7:** Negative changes in financing attitude toward served industries before/after the pandemic

	Trade	Transportation	Culture	Energy	Agriculture	Construction
Before the pandemic	8.48	0.98	1.01	2.42	3.56	2.98
During and after the pandemic	7.94	1.04	0.94	2.16	3.38	2.27
Changes	*−*6.37%	*−*0.89%	*−*6.93%	*−*10.79%	*−*4.96%	*−*23.83%
*p*-value	0.065^*^	0.742	0.224	0.074^*^	0.275	0.000^***^

*Notes*. The *p* values in the last line refer to the differences in FSPs’ financing attitude to each industry before and after the pandemic. The values in this table refer to the weighted score of the corresponding items in the Appendix of each type, reflecting the importance of each industry. The higher the value, the higher is the degree of importance. The weighted score of each item = ∑(*frequency* times *weight*)/*the number of observations*; the *weight* is determined by where the items are arranged. For example, if there are three items involved in sorting, then the item in the first position gets a weight of 3, the one in the second position gets a weight of 2, and the one in the third position weights 1

*** *p <* 0.001, ** *p <* 0.01, * *p <* 0.1

These results reflect the general changes in FSPs’ risk preferences. The medical industry has received much attention and support as it is fighting the pandemic directly. However, FSPs should intensify the risk control of loans and avoid overinvestment. Similarly, the public service industry contributes to ensuring public production and order, and thus also receives more financial support. New information and communication technologies have contributed in response to the pandemic and show development potential. The business service industry has marginally suffered due to the pandemic because most businesses can be operated online. Thus, both the IT and business service industries can obtain support from FSPs. Notably, positive changes can be seen in the hospitality and tourism industry, though it has lost numerous orders and has stagnated due to the pandemic. One possible explanation is that most FSPs believe that the relevant demand will rebound sharply at the end of the pandemic.

However, it will be harder for SMEs in the construction industry to obtain financing from FSPs. The supply chain in the construction industry is relatively long and sophisticated, causing longer cash-to-cash cycles for SMEs, and this situation has worsened due to the pandemic. The significant increase in lending risks has left FSPs reluctant to lend capital to SMEs in this industry. Moreover, some labor-intensive industries, such as the culture and agriculture industries, have been negatively affected by isolation and transportation disruption. The dramatic reduction in the energy demand has made financing difficult, as evidenced by the recent drop in oil prices. The trade industry has also suffered significantly. SMEs in these industries that are affected seriously by the pandemic may not obtain sufficient financial support from FSPs, leading to a new “disaster” for them.

#### Changes in financing products

We explore whether the FSPs adjust their financing products due to the COVID-19 pandemic. Based on the types of financing business and risk control, we classify fixed asset mortgages and the third-party guarantees as “traditional” financing products, and others such as goods or inventory receipt pledged, factoring, and financing with letter of credit (LC) as “nontraditional.”

As shown in Table 8, the weighted score of fixed asset mortgage increases significantly (8.06%; *p* = 0.025 < 0.1), while the third-party guarantee has no significant change. These results indicate that fixed asset mortgages receive more attention from FSPs because of its relatively strict risk control. However, third-party guarantees may not be preferred due to uncertainty and increased systematic risk. However, such traditional financing products cannot effectively meet the demands of SMEs without sufficient fixed assets, though for most FSPs, it is relatively easy to control the financing risk of the traditional products.

**Table 8:** Changes in “traditional” financing products before and after the pandemic

	Fixed asset mortgage	Third-party guarantee
Before the pandemic	4.20	4.08
During and after the pandemic	4.54	4.07
Changes	8.06%	−0.18%
*p*-value	0.025^*^	0.861

*Notes*. *** *p <* 0.001, ** *p <* 0.01, * *p <* 0.1

The analysis toward nontraditional financing products also provides meaningful results, shown in Table 9. There is a tendency to increase the provision of almost all nontraditional financing products. Among them, financing with insurance (68.7%; *p* < 0.001), financing based on taxation data analysis (31.76%; *p* = 0.017 < 0.1), and financing based on operational data mining (18.97%; *p* = 0.004 < 0.01) have significant positive changes. This indicates that risk-sharing through collaboration between FSPs and insurance and the role of critical data such as tax and operational data is emphasized by FSPs. These three products are not yet part of the major business of FSPs, which may be developed continuously with greater potential.

**Table 9:** Changes in “nontraditional” financing products before and after the pandemic

	Goods/inventory pledged	Trade agency	Factoring	Financing based on taxation data analysis	Financing with insurance	Financing based on operational data mining	Financing with LC
Before the pandemic	3.86	1.63	4.22	0.86	0.96	2.36	1.13
During and after the pandemic	4.19	1.74	4.25	1.13	1.625	2.81	1.14
Changes	8.48%	7.01%	0.61%	31.76%	68.70%	18.97%	1.30%
*p*-value	0.168	0.787	0.638	0.017^*^	0.000^***^	0.004^***^	0.933

The *p* values in the last line refer to the differences in FSPs’ financing attitude to each industry before and after the pandemic. The values in this table refer to the weighted score of the corresponding items in the Appendix of each type, reflecting the importance of each industry. The higher the value, the higher is the degree of importance. The weighted score of each item = ∑(*frequency* times *weight*)/*the number of observations*; the *weight* is determined by where the items are arranged. For example, if there are three items involved in sorting, then the item in the first position gets a weight of 3, the one in the second position gets a weight of 2, and the one in the third position weights 1

*Notes*. *** *p <* 0.001, ** *p <* 0.01, * *p <* 0.1

## Conclusions, suggestions, and limitations

### Conclusions

This study proposes a theoretical framework based on expectancy theory to explore how FSPs support SMEs in the context of the COVID-19 pandemic. Specifically, we investigate multi-level factors that influence the FSPs’ expectancy and valence. We also explore the differences in these factors between commercial banks, non-bank financial institutions, and credit-enhanced FSPs.

Overall, under the impact of the pandemic, many factors at different levels have lowered FSPs’ expectancy of financing SMEs. Among these factors, insufficient financial resources and informatization capabilities are common challenges for some FSPs, and lack of coordination with focal firms is a challenge for nearly half of them. Besides, most FSPs have claimed strong support from peer FSPs like commercial banks and insurance companies. Most FSPs ask for the support to access public data from public administrations at the institution level, allowing online due diligence by regulators and increasing liquidity. Furthermore, these factors do not impact the three types of FSPs equally. Commercial banks are primarily concerned about SMEs’ data, expecting to improve their informatization capability and collaboration with focal firms. They also seek institutional support, especially from public administrations and regulators. However, credit-enhanced FSPs are in greater need of capital, increasing liquidity, and support from peer FSPs. However, they have comparative advantages in informatization capability and collaboration with focal firms. Thus, to enhance FSPs’ financing expectancy in such a turbulent time, broader and deeper collaboration among the multi-stakeholders is needed (Ntwiga 2020; Wu et al. 2019). Moreover, the collaboration between FSPs, public administrations, and regulators also matters, especially under the pandemic’s impact. The result of this study has indicated that FSPs demand deeper collaboration with the institutional participants to promote diversified information sharing like public data, and for flexible decision-making and operation, such as increased liquidity and risk tolerance. Consequently, in the face of external shocks like the pandemic, it is necessary to explore innovative financing modes through multilateral collaboration.

According to the analysis of FSPs’ financing valence, we establish how FSPs make a trade-off decision between economic performance and social responsibility. Most of the surveyed FSPs actively undertook social responsibility in financing SMEs, echoing some existing studies (Talbot and Ordonez-Ponce 2020). Most FSPs maintain or increase their SMEs’ financing at a relatively lower interest rate despite the increase of default rates, beneficial for numerous SMEs for fundraising. Commercial banks provide more capital with lower interest rates than others, followed by non-bank financial institutions. Moreover, nontraditional financial businesses such as financing with insurance and financing based on data mining or taxation analysis are conducted, reflecting that FSPs increase financing by adopting innovative financing modes and emerging technologies. The strategic adjustments make it more accessible for SMEs to receive funding at low costs, while also increasing the burden on FSPs.

However, in addition to the motivation for social responsibility, FSPs have expressed strong concerns about economic performance, reflected in the different levels of financing willingness toward diverse industries. FSPs increase financing to SMEs in the medical industry and public service industry while reducing the level of financing to SMEs in the construction, energy, and trade industries. Industries that have better prospects attract FSPs for financing activities. However, those severely affected by the pandemic require more financial support, while FSPs are unwilling to provide enough financing for the higher default risk (Gong et al. 2020). Thus, these issues indicate that SMEs’ financing problems cannot be solved by FSPs alone, and more collaboration between FSPs and other stakeholders is required.

### Suggestions

The results and analyses have several implications for SMEs, public administrations, regulators, and FSPs.

SMEs must recognize the strategic adjustments of FSPs and take corresponding measures. First, though most FSPs provide financial support proactively, this behavior may not benefit SMEs in some industries. SMEs in the construction, energy, and trade industries may still be confronted with capital shortages. Therefore, SMEs in these industries need to focus on cash flow management. Second, SMEs should enhance their information sharing with FSPs and their collaboration with focal firms. FSPs focus on the operational data of SMEs and care about their business development and risk control under the impact of the pandemic. Most businesses are conducted based on collaborations with focal firms, including financing with goods or warehouse receipt pledging and factoring.

Public administrations and regulators can formulate policies that constitute support to FSPs, including access to public data, permitting online due diligence, and restricting the late payments between firms; however, differentiated support can effectively benefit FSPs. Specifically, encouraging focal firms to cooperate and deal with bad debts will promote commercial banks to serve SMEs effectively. Commercial banks and non-bank financial institutions may also require exemption of defaults by affected debtors. Moreover, regulators should provide more support for credit-enhanced FSPs by reducing required reserve ratios, relaxing the restriction of risk tolerance of commercial banks and non-bank financial institutions, and allowing online accounts to be opened for commercial banks. Further, public administrations and regulators can consider providing extra financial support such as fiscal subsidies to SMEs in some industries that have been affected seriously and have difficulty in accessing financing services from FSPs.

It is evident that there are advantages for the different types of FSPs; specifically, commercial banks have a greater need to improve their informatization capabilities, and credit-enhanced FSPs, including fintech companies, could provide relevant support through the application of advanced information technology, analysis, or credit investigation. Similarly, credit-enhanced FSPs are under the restriction of insufficient funds and lending, an advantage of commercial banks and non-bank financial institutions. Therefore, cooperation between them could effectively enhance SMEs’ financing activities. Additionally, cooperation with diversified FSPs such as insurance can effectively reduce financing risks, especially during the COVID-19 pandemic.

### Research limitations

This study has several limitations. First, the sample in this research is limited, and the results do not apply to all kinds of FSPs, though conducting surveys among FSPs is relatively more difficult. Second, the sampling time is in the early period of the pandemic. Some FSPs need more time to adjust their strategies. Third, none of the financing expectancy, financing valency, or financing of SMEs is measured in this research; the relationships between them and the antecedents could be tested empirically in future studies. Fourth, we only classify FSPs into three types according to the presence or absence of financial business licenses. In the future, however, we aim to explore each type of FSP’s distinctive behaviors or attitudes toward the pandemic in detail. For example, fintech companies and logistics service providers may differ in their financing strategies and need for external support, though they are both credit-enhanced FSPs.

## Data Availability

The datasets used during the current study are available from the corresponding author on reasonable request.

## Appendix

**Table 10:** Development process and content of the survey

Indicators	References	Expert scrutiny	Questions & Items	Requirements
Sample characteristics				
Total assets in the last fiscal year	*Standard Provisions on the Classification of Financial Enterprises* from the People’s Bank of China	Adopt	What are the total assets of the company in the last fiscal year? (in RMB) A. < 0.1 billion; B. 0.1*–*1 billion; C. 1*–*2 billion; D. 2*–*5 billion; E. 5*–*20 billion; F. 20*–*40 billion; G. 40*–*100 billion; H. 100*–*500 billion; I. 500*–*4000 billion; J. > 4000 billion	Single choice
Financing volume to SMEs in the last fiscal year	Gomm 2010; Lu et al. 2020	To design the specific scale based on professional experience	What are the financing volume to SMEs in the last fiscal year? (in RMB) A. < 0.1 billion; B. 0.1*–*1 billion; C. 1*–*5 billion; D. 5*–*10 billion; E. 10*–*20 billion; F. 20*–*50 billion; G. 50*–*100 billion; H. > 100 billion	
The number of SMEs served in the last fiscal year		Considering that the number of SMEs may influence FSPs’ decisions to provide financing, it is necessary to investigate this characteristic	How many SMEs has your company served in the last fiscal year? A. < 100; B. 100*–*1000; C. 1000*–*10,000; D. 10,000*–*50,000; E. 50,000*–*100,000; F. 100,000*–*500,000; G. > 500,000	
The ownership of main clients (SMEs served)	Zhu et al. 2020	The pandemic impacts differently for different ownership of clients, which will be similarly taken into FSPs’ consideration	What is the ownership of the main client SMEs you have served? A. State-owned; B. Private; C. Foreign or Sino-foreign joint	
Business types	Martin and Hofmann 2017; Song et al. 2018; Xu et al. 2018	In combination with classification in practice, experts have added more business types	What is the business type of your company? A. Commercial bank; B. Guarantee company; C. Factoring company; D. Small-loan company; E. Fund company; F. Financial holding company; G. Trust company; H. Supply chain service provider; I. Fintech service provider; J. Industrial internet platform; K. Logistics service provider; L. Foreign trade service provider; M. Internet finance company; N. Others	
Financing expectancy				
Lack of internal resources and capabilities	Abbasi et al. 2018; Beck 2013	In combination with practical experience, experts have independently listed the relevant example at first. Then, we select the most representative statements to develop the specific item in each indicator. The item will be adopted only if all the experts agree	Which of the following item is in line with the company’s current situation? A1. Lack of enough technical talents and business professionals A2. Lack of enough financial resources (like money, etc.) A3. Lack of enough informatization capacity	Multiple choice
Lack of focal firms’ incoordination	Lekkakos and Serrano 2016; Wu et al. 2019		B1. Focal firms are unwilling to cooperate B2. Focal firms have big problems on their own	
Supports from peer FSPs	Ntwiga 2020; Zhu et al. 2015		C1. Cooperate with insurance companies C2. Cooperate with other banks and trusts	
Supports from public administrations	Zhang et al. 2020; Zhu et al. 2020		D1. Open public data D2. Encourage focal firms to cooperate D3. Limit the delay in payments among firms D4. Assistance in dealing with debts caused by the pandemic D5. Exemption of default by the affected debtor D6. Reduce some taxes on financial institutions	
Supports from regulators	Zhu et al. 2020		E1. Allow online due diligence E2. Increase the liquidity of financial institutions E3. Allow the increase of risk tolerance E4. Allow online account opening	
Financing valence				
Changes of financing volume	Gomm 2010; Lu et al. 2020; Ongore and Kusa 2013	Adopt	How will the financing volume to SMEs change in your company during/after the pandemic? A. Comprehensively decrease B. Most decrease, some increase C. Remain unchanged D. Most increase, some decrease E. Comprehensively increase	Single choice/ 5-point Likert Scale
Changes of financing rate	Gomm 2010; Lu et al. 2020		How will the financing rate change during/after the pandemic? A. Comprehensively decrease B. Most decrease, some increase C. Remain unchanged D. Most increase, some decrease E. Comprehensively increase	
Changes of default rate	Ongore and Kusa 2013		How will the default rate change during/after the pandemic? A. Comprehensively decrease B. Most decrease, some increase C. Remain unchanged D. Most increase, some decrease E. Comprehensively increase	
Served industries before the pandemic	Bartik et al. 2020; Zhu et al. 2020; *Guidelines on Industry Classification of Listed Companies* from China Securities Regulatory Commission	Adopt	Before the pandemic, to which industry groups are the SMEs that you provided financing services belong? (Arrange them in order from primary to secondary. Industries not covered need not be included) A. IT; B. Business service; C. Education; D. Manufacturing; E. Trade; F. Medical industry; G. Hospitality and Tourism; H. Transportation; I. Culture; J. Energy; K. Public service; L. Agriculture; M. Construction	Ranking
Served industries after the pandemic			After the pandemic, to the SMEs of which industry groups will your company mainly provide financing services? (Arrange them in order from main to secondary. Industries not covered need not be included) A. IT; B. Business service; C. Education; D. Manufacturing; E. Trade; F. Medical industry; G. Hospitality and Tourism; H. Transportation; I. Culture; J. Energy; K. Public service L. Agriculture; M. Construction	
Financing products before the pandemic	Lekkakos and Serrano 2016; Silvestro and Lustrato 2014; Song et al. 2018; Xu et al. 2018	In combination with practical experience, experts have independently listed the relevant financing products at first. Then, we remove duplicate ones. The item will be adopted only if all the experts accept	Before the pandemic, which financing products or services are mainly provided by your company? (Arrange them in order from main to secondary. Business not provided need not be included) A. Fixed asset mortgage; B. Third-party guarantee; C. Goods/inventory pledged; D. Trade agency; E. Factoring; F. Financing based on taxation analysis; G. Financing with insurance; H. Financing based on data mining; I. Financing with LC	
Financing products after the pandemic			After the pandemic, which financing products or services will be mainly provided by your company? (Arrange them in order from main to secondary. Business not provided need not be included) A. Fixed asset mortgage; B. Third-party guarantee; C. Goods/inventory pledged; D. Trade agency; E. Factoring; F. Financing based on taxation data analysis; G. Financing with insurance; H. Financing based on operational data mining; I. Financing with LC	

## Abbreviations

FSPs: Financial service providers
SMEs: Small and medium enterprises
